# The caloric and sugar content of beverages purchased at different store-types changed after the sugary drinks taxation in Mexico

**DOI:** 10.1186/s12966-019-0872-8

**Published:** 2019-11-12

**Authors:** Lilia S. Pedraza, Barry M. Popkin, Carolina Batis, Linda Adair, Whitney R. Robinson, David K. Guilkey, Lindsey Smith Taillie

**Affiliations:** 1 Department of Nutrition, Carolina Population Center, Gillings School of Public Health, University of North Carolina at Chapel Hill, CB # 2107 Carolina Square, Chapel Hill, NC 27516-3997 USA; 2 Center for Nutrition and Health Research, National Institute of Public Health, Cuernavaca, Morelos Mexico; 3 Department of Epidemiology, Gillings School of Public Health, University of North Carolina, Chapel Hill, North Carolina USA; 4 Economics Department, University of North Carolina, Chapel Hill, North Carolina USA

**Keywords:** SSBs tax, Mexico, Calories, Sugar, Food-stores

## Abstract

**Background:**

Following the 2014 sugary drinks tax implementation in Mexico, promising reduction in the volume of purchases of taxed beverages were observed overall and at different store-types. However, the tax’s effects on purchasing patterns of calories and sugar remain unclear.

**Methods:**

Using longitudinal data from Mexican households (*n* = 7038), we examined changes in volume, calories and total sugar of packaged beverages purchased from 2012 to 2016 overall and by store-type. We used fixed effects models to estimate means for volume, calories, and sugar of households. To address the potential selectivity from households shopping at different stores, we calculated inverse probability weights to model the purchases changes over time by store-type.

**Results:**

For taxed beverages, the volume of purchases declined by − 49 ml and -30 ml in the first year and second year post tax (2014 and 2015, respectively), while purchases leveled off in the third year of the tax (2016). Calories and sugar from taxed beverage purchases decreased over time, with the majority of the declines occurring in the first two years post-tax implementation. The volume of untaxed beverage purchases increased, whereas changes in calories and total sugar of untaxed beverages were minimal. Store level purchases of taxed beverages significantly decreased in the first two years post taxation (2014 and to 2015) only in supermarkets and traditional stores. The steepest declines in purchases of taxed beverages in 2014 were observed at supermarkets (− 40 ml or − 45%). The volume of purchases of untaxed beverages increased over time in almost all store-types, while calories and sugar minimally decreased over time.

**Conclusion:**

Although the Mexican tax on SSBs has lowered the purchases of sugary drinks 3 years after the tax implementation, the tax should be strengthened and store-specific interventions should be implemented to further reduce SSBs purchases in the Mexican population.

## Introduction

The intake of sugar-sweetened beverages (SSBs) is associated with increased risk of overweight and obesity, type two diabetes, stroke, and mortality from cardiovascular disease [1–4]. In Mexico, where overweight and obesity is present in 72% of adults, and in 33 and 36% of school-age children and adolescents, respectively, SSBs are the main source of added sugars. In fact, SSBs contribute 10% of the overall energy intake [5–7] Policies like SSB taxes are considered effective because they affect millions of people at once rather than seeking to change individual behaviors [8]. Hence, in January 2014, a one MXN peso per liter (≈10%) tax on SSBs was enforced in Mexico as an effort to prevent further increases in obesity. Following the tax implementation, the volume of household purchases of taxed beverages fell by an average of 8% over two years [9] suggesting a promising impact on the population’s food-purchasing choices. Because taxed beverages contribute to 6% of the overall energy intake in the Mexican population [10], the reduction of caloric and sugar intake were the main goal of the tax and are key for obesity prevention. However, the tax’s effects on purchases of calories and sugar is still unknown.

In addition, while previous studies have shown that changes in the volume of taxed products were greatest for low-income and high-consuming households [9, 11, 12], little is known about how the purchasing patterns and purchases of calories and sugars of taxed beverages changed at different store types. Because food suppliers are major mediators between the food environment and the eating behaviors that influence the development of obesity [13, 14], determining whether calories and sugars from beverages vary by store-type is important. Evidence has shown that the types of beverages that Mexican households purchase vary by store-type and purchases of taxed beverages are highest at traditional stores [15]. Store-level interventions and policies can potentially increase the healthfulness of stores’ assortment of foods and beverages, improve households’ diet quality and prevent obesity [16–18]. A clear understanding of the nutrient profile of beverages purchased at different Mexican store-types as affected by the SSBs tax can provide evidence to design targeted interventions to improve purchasing choices.

To address these gaps, this research examined changes in volume, calories and total sugar of packaged beverages purchased from 2012 to 2016 using longitudinal data from urban Mexican households. We present these results overall and by store-type, considering the sociodemographic characteristics influencing households’ probability to shop at a particular store-type. Using purchasing data from before and after the implementation of the sugary drinks tax in Mexico allowed us to assess long-term changes in the nutrient profile of beverage purchases and purchasing choices associated with the SSBs tax.

### Data

#### Purchasing data

This study used household-level data on the volume (milliliters), kilocalories and total sugar (grams) of packaged beverages purchases from The Nielsen Company’s Mexico Consumer Panel Services (Nielsen CPS) database for years 2012–2016. The 5-year analytical sample includes 338,187 household-month observations from 7038 households drawn from urban areas with > 50,000 inhabitants in Mexico. The average follow-up time of households was 48 months. In accordance with the Mexican SSBs tax policy, the time-period January 1, 2012- December 31, 2013 was considered the pre-tax period, while January 1, 2014-December 31, 2016 was considered the post-tax period.

Nielsen CPS detailed data collection process has been described elsewhere [9, 11, 15, 19, 20]. Briefly, Nielsen CPS interviewers gather purchasing information from packaged products with a barcode through bimonthly household audits, in which enumerators visit participants’ homes and retrieve information from receipts and logs from purchasing diaries, conduct pantry inspections and re-scan barcodes from available products.

#### Nutritional information

The packaged beverages available from Nielsen CPS were linked to the UNC Mexican Nutrition Fact Panel (MxNFP) dataset that contains nutrition information for packaged products available in the Mexican food supply.

The MxNFP is a composite database created by the University of North Carolina (UNC) using four data sources. From the Mexican National Institute of Public Health (Instituto Nacional de Salud Pública, INSP), records were available through the collection of product photos taken in stores following the International Network for Food and Obesity/non-communicable diseases Research, Monitoring and Action Support (INFORMAS) framework [21, 22]. The nutrition facts from the photos taken were recorded into Excel or the Redcap platform [23] and available for 2014–2016. From Mintel Global New Product Database [24], a commercial database that monitors the introduction of new products into the North American and Latina American markets, nutrition facts information was available for 1996 to present. From Product Launch Analytics (Datamonitor, PLA) [25], a global web-based dataset of newly launched consumer products, nutrition facts records were available from January 2009 to May 2010. Finally, from Chile RedCap, a Chilean dataset with nutritional information collected from photos taken in stores of Latin-American products and recorded into the Redcap platform, records were available for 2015–2016.

Products were linked from the MxNFP to the household purchase data at the barcode level and then individually reviewed by a team of registered dietitians (RD) for nutritional accuracy. A direct barcode-to-barcode match between the MxNFP and Nielsen CPS products was found for 93% of purchases. If a direct match was found between an MxNFP record and a Nielsen CPS product, the most recent NFP record was chosen. Only one MxNFP record was selected as match for each Nielsen CPS product during the 5-y period. If a direct barcode match was not found, then the product was matched to the MxNFP record of a product with the closest description and with the highest sales. The INSP contributed 44% of the MxNFP, Mintel contributed 56% of the MxNFP information, and PLA and Chile RedCap contributed less than 1% of the MxNFP data.

#### Product categorization

Trained dieticians reviewed and grouped each unique beverage product in Nielsen CPS into taxed and untaxed categories according to the Mexican legislation, which specifies that all beverages with added sugar have a 1 peso per liter tax (~ 10%). Additional file 1: Table S1 presents a detailed description of the taxed and untaxed beverages included in this study, including beverage sub-categories (e.g., carbonated soft drinks, juices and fruit drinks, etc.).

For this research, all weight information in grams provided by Nielsen CPS for beverages mixes in dry form (e.g. instant coffee and dried milk) was reconstituted into liquid form (milliliters) using standardized conversion factors reflecting packaging instructions.

#### Store-type categorization

The Nielsen CPS data includes information on the retailer where every shopping episode occurs. As in previous work [15], stores were categorized using store size, product assortment and additional services provided to consumers [26, 27] into convenience stores (e.g. 7-eleven), supermarkets (e.g. Walmart), wholesalers (e.g. Costco), traditional stores (usually attended by the owner, including stores installed in permanent public markets), and others (e.g. department stores, pharmacies, movie theaters, etc.).

In Mexico, it is common for households to have 20 liter-jugs of drinking water delivered to the house, making “home-delivery” an additional source of untaxed beverage purchases. However, this study primarily focused on changes that occurred in consumer purchasing choices of caloric and sugary beverages in stores before and after the tax. Thus, although home-delivery was included in the analytical models for store-types, this study provides descriptive and predicted data on home-delivery (Additional file 3: Table S3 and Additional file 5: Table S5), but does not include home-delivery in the results figures (Fig. 2).

## Methods

### Covariates

Household-level covariates included household composition (total number, age and sex of all household members) and household socio-economic status (SES). Consistent with previous studies using the Mexico Nielsen CPS data, SES was categorized into low, middle and high. The SES index is comprised of seven household assets (number of rooms, type of floor, number of bathrooms, shower, gas range, number of light bulbs and number of cars), and the education level of the head of the household [9, 11, 15, 19, 20].

Additional covariates included geographic area-specific daily minimum wage [28], and state-quarterly unemployment rates and consumer price index [29, 30] as contextual factors to control for spending power and cost of living across the country over the 5-y study period.

Nielsen CPS provides household weights that are based on household composition, city, and socioeconomic measures and vary annually, which we included in our analyses to support the generalizability of our results to all urban Mexican households.

### Statistical analyses

We estimated weighted and adjusted means for volume, calories, and sugar at the household-monthly level over time, overall and by store type. We used household-level fixed effects models adjusted for time-varying SES, household composition and contextual factors to predict the changes over time in volume (milliliters/capita/day), calories (kcal/capita/day) and total sugars (grams/capita/day) of taxed and untaxed beverage purchases. We used fixed effects at the household level to control for unobserved time-invariant household characteristics that influence beverage purchasing (e.g. beverage preferences) and that are correlated with some of the observed explanatory variables, which would cause random effects models to be biased [31]. We included dummies for month of survey to adjust for seasonality. We performed sensitivity analyses specifying a range of simpler to more complex fixed effects models that included pre-and post- taxation dummy variables (e.g. 2012–2013==0; 2014–2016 = 1), time trends, and quadratic time trend terms interactions with year to check for robustness in results. Results from all models were comparable, thus we chose the simplest model to allow better interpretability.

### Store-type analyses

An array of factors including both store-type (e.g. product assortment, prices, proximity, etc.) and household attributes (i.e. preferences, sociodemographic characteristics, etc.) influence the store-type where households choose to shop [32, 33]. Thus, the nutrient profile of beverage purchases might be explained not by the quality of the products a store offers, but by the characteristics of the customers it attracts [34]. In our sample, household beverage purchases at different store-types varied by their socioeconomic status, number of members, members’ age, and members’ sex, among other characteristics. To model the changes over time in volume, calories and total sugar of taxed and untaxed beverage purchases by store-type, we needed to account for the sample being different by store type conditional to their sociodemographic characteristics. Similar to previous papers analyzing trends by store-type, we used inverse probability weights to address the potential selectivity from households shopping at different stores [34, 35]. First, we predicted the probability of being a beverage shopper at each store-type using logistic regressions weighted with the Nielsen CPS sampling weights, and adjusted by covariates associated with purchasing choices (i.e. SES, household size and composition, minimum wage, unemployment rate and consumer price index). Then, we used the predicted probabilities to calculate store-specific inverse probability weights for each year, to account for the probability of being a shopper at each store-type. Finally, we used the inverse probability weights in fixed effects regressions to obtain pre-and post-tax volume and nutrient content means of purchases by store-type [36, 37]. By using these weights in each store-type model, we created a balanced distribution of covariates between households that purchased beverages at a specific store-type and those who did not.

All statistical analyses were performed using Stata version 14 (StataCorp, College Station, TX, USA) and statistical significance was set at a *p*-value ≤0.05 using the Bonferroni method to account for multiple comparisons.

## Results

The sociodemographic characteristics of the Nielsen CPS households from 2012 to 2016 are shown in Additional file 2: Table S2 and were consistent over time. During the 5-y study period, households were predominantly in the middle SES (54%), households were mostly composed of between four to five members (41%) and had between two to three children between the ages 0–19 (44%). The weighted unadjusted mean volume, kilocalories and total sugar of taxed and untaxed beverages per capita/day, overall and by store-type in 2012 and 2016 are shown in Additional file 3: Table S3. On average, in 2016, households purchased 77% (148 ml) of taxed beverages at traditional retailers. In 2016, households purchased 58% (523 ml) of untaxed beverages through home-delivery, followed by 17% (156 ml) and 16% (148 ml) of untaxed beverages at other retailers and traditional retailers, respectively. Traditional stores contributed 83% to purchases of taxed beverages calories and sugar (57 Kcal, and 14 g, respectively), in 2016 (percentages estimated from Additional file 3: Table S3 data).

### Changes in volume, kilocalories and total sugar of beverage purchases

Predicted adjusted mean volume, kilocalories and total sugar of taxed and untaxed beverage purchases per capita/day for 2012–2016 and their absolute and relative differences between years are presented in Additional file 4: Table S4.

**Fig. 1:**
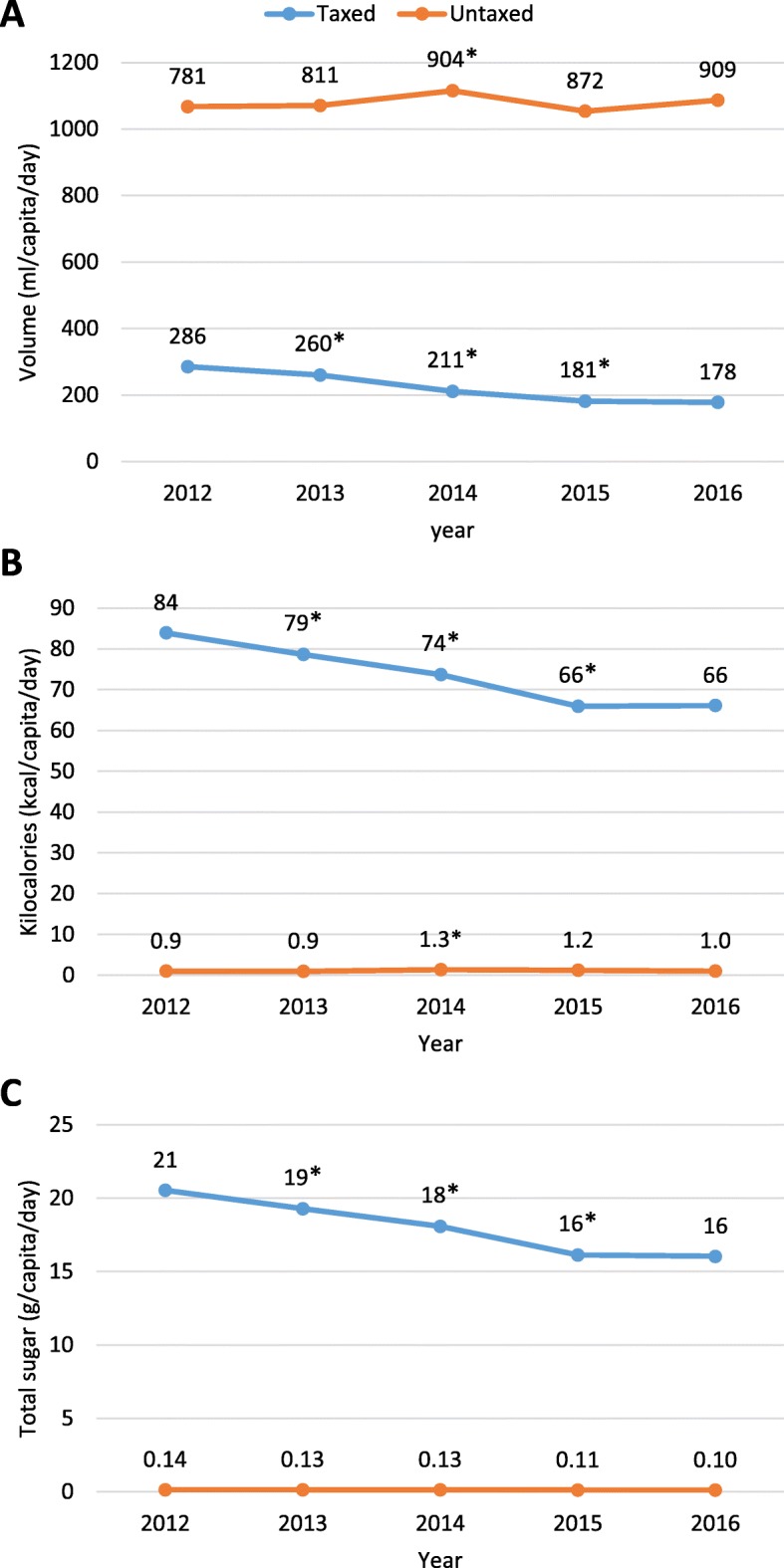
Daily per capita (**a**) volume, (**b)** Kilocalories, and (**c**) total sugar of taxed and untaxed beverage purchased by urban Mexican households. Source: Authors’ own analyses and calculations based on data from Nielsen through its Mexico Consumer Panel Service (CPS), for the beverage categories for January 2012 – December 2016. The Nielsen Company, 2016. Nielsen is not responsible for and had no role in preparing the results reported herein. Volume, kilocalories and total sugar means of taxed and untaxed beverage purchases obtained using fixed effect models adjusted by socioeconomic index, household size and composition, minimum wage, unemployment rate and consumer price index, and weighted to be representative of populations in areas with more than 50,000 inhabitants. *p-value < 0.05 comparing with previous year using the Bonferroni method to account for multiple comparisons

Figure 1 shows the adjusted daily per capita means of volume, kilocalories and total sugar for taxed and untaxed beverages purchased from 2012 to 2016. For taxed beverages, the volume of purchases decreased steadily over time. However, the size of the reductions changed over time, such that the largest reductions were observed in the first and second year of the tax (2014 and 2015), before leveling off in the third year of the tax (2016). Specifically, the largest absolute and relative decline (− 49 ml and-19%, respectively, *p* < 0.05) of taxed beverage purchases was observed from the last pre-taxation year (2013) to the first post taxation year (2014). From the first to the second year post-tax (2014 to 2015), there was a significant decline of − 30 ml (− 14%, *p* < 0.05) in the purchases of taxed beverages, whereas the difference in volume purchases of taxed beverages from the second to the third year post-tax (2015 to 2016) was non-significant (− 3 ml, − 2%).

Calories and sugar from taxed beverage purchases also decreased over time and followed a similar pattern in which the majority of the declines occurred in the first two years post-tax implementation. From the last year pre-tax (2013) to the first year post-tax (2014) purchases of taxed beverages declined by − 5 kcal and -1 g of sugar (a relative decline of − 6% for both, *p* < 0.05). Then, the decline in taxed beverages increased from the first to the second year of post-tax (2014–2015) (− 8 kcal and − 2 g of sugar or an-11% relative reduction for both, *p* < 0.05). No significant changes in calories and sugar were observed between the second to the third year post-tax (2015 to 2016).

For untaxed beverages, the volume of purchases increased over time, with a significant increase of 93 ml (11%, p < 0.05) observed only between the last year pre-tax and the first year post tax (2013 to 2014) (Fig. 1). Contrary to volume, the changes in calories and total sugar of untaxed beverages were minimal.

### Volume, kilocalories and total sugar trends by store-type

**Fig. 2:**
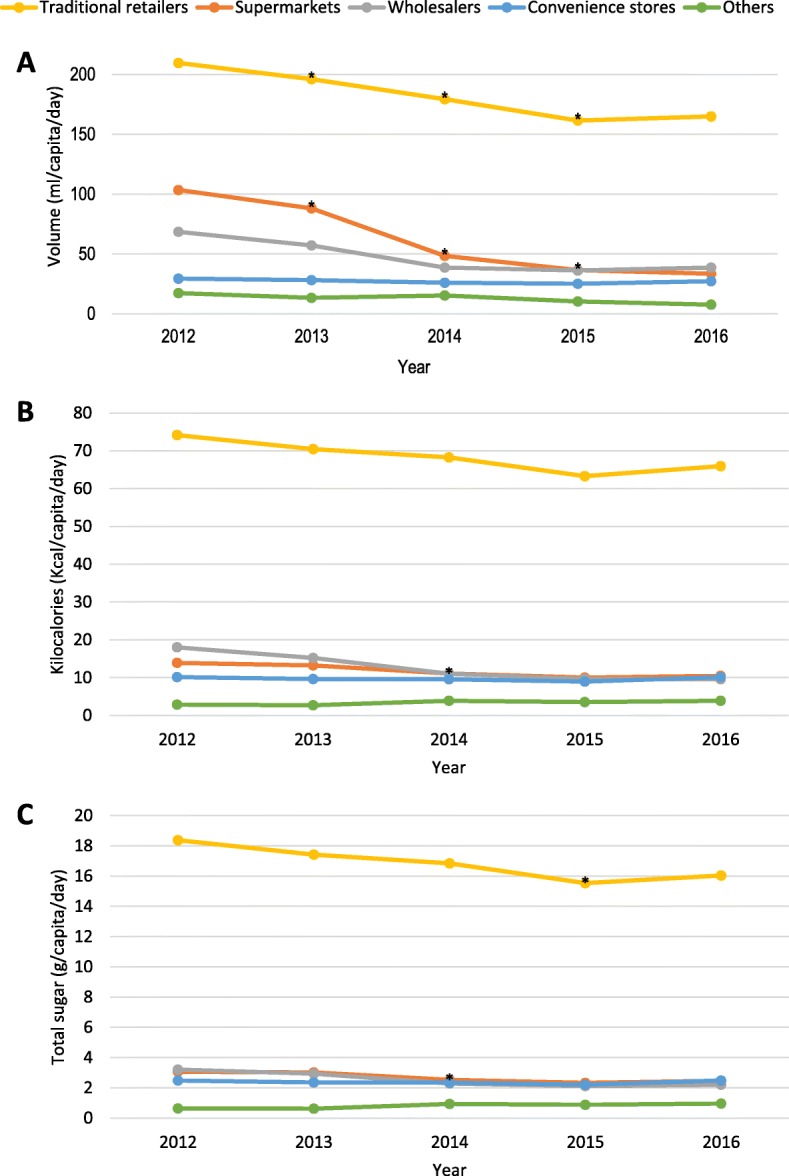
Daily per capita (**a**) volume, (**b**) Kilocalories, and (**c**) total sugar of taxed beverages purchased by urban Mexican households at different store-types. Source: Authors’ own analyses and calculations based on data from Nielsen through its Mexico Consumer Panel Service (CPS), for the beverage categories for January 2012 – December 2016. The Nielsen Company, 2016. Nielsen is not responsible for and had no role in preparing the results reported herein. Volume, kilocalories and total sugar means of taxed and untaxed beverage purchases obtained using store-type specific fixed effect models using inverse probability weights and adjusted by socioeconomic index, household size and composition, minimum wage, unemployment rate and consumer price index, and weighted to be representative of populations in areas with more than 50,000 inhabitants. *p-value < 0.05 comparing with previous year using the Bonferroni method to account for multiple comparisons

Figure 2 shows the daily per capita volume, kilocalories and total sugar for taxed beverages purchased from 2012 to 2016 by store-type, weighted by the inverse probability of purchasing at different store-types. Predicted mean volume, kilocalories and total sugars (per capita/day) of taxed and untaxed beverages purchased by store-type from 2012 to 2016 and their absolute and relative differences between years are presented in Additional file 5: Table S5.

As observed in the total population results, the volume of taxed beverage purchases by store-type decreased over time, except for convenience stores, where purchases of taxed beverages remained unchanged. However, the decreases in the purchases of taxed beverages in the first two years post taxation (2014 and to 2015) were statistically significant only in supermarkets and traditional stores. In the first year post tax (2014), the purchases of taxed beverages at traditional stores declined by − 17 ml (− 9%), however, the steepest absolute and relative declines in purchases of taxed beverages in the same year were observed at supermarkets (− 40 ml and − 45%, respectively).

Calories and total sugar of taxed beverage purchases declined over time across store-types, except for convenience stores where calories and sugar of taxed beverages remained unchanged, and other retailers where total sugar of taxed beverages remained the same over time.

For untaxed beverages, the volume of purchases increased over time in all store-types and through home-delivery, except for convenience stores where purchases of untaxed beverages decreased from 2012 to 2016. In general, calories and total sugar from untaxed beverages in all store-types decreased over time, but the absolute size of the changes were minimal.

## Discussion

To our knowledge, this is the first study to look at changes in volume, calories and sugar from beverages overall and by store type in urban Mexican population, from two years before (2012–2013) to three years after (2014–2016) the SSBs tax implementation.

Overall, we observed a reduction of 108 ml (− 37%), 18 Kcal (− 21%) and 5 g of sugar (− 23%) from purchases of taxed beverages between 2012 and 2016. Our results showed a sustained and significant decrease in the volume, calories and sugar purchases from taxed beverages in the first two years after the tax (2014–2015). However, this trend plateaued 3 years after the tax, with very little change between 2015 and 2016. Meanwhile, purchases of untaxed beverages significantly increased only in the first year after the tax (2014). Our store-type specific results were consistent with previous findings, showing that Mexican households purchase the most volume, calories and sugars from taxed beverages at traditional stores [14]. Although significant declines in purchases of taxed beverages in the first two years post taxation (2014 and 2015) were observed in traditional stores and supermarkets, the latter store-type showed the steepest absolute and relative declines in the first year post tax (2014).

Our overall results were expected since the observed decreases in the purchases of taxed beverages and increase in purchases of untaxed beverages immediately after the implementation of the tax are consistent with previous findings in the Mexican population [9, 11].

It is important to note that our results show that the Mexican tax on SSBs achieved its intended goal of lowering the purchases of sugary beverages even 3 years after the tax implementation. That is, that the volume of purchases was still lower in the third post taxation year (2016) compared to the pre taxation years (2012–2013). The effect of the tax on the population purchasing choices is also reflected in the reduction in calories and sugars from taxed beverages purchases. However, although we saw a sizeable relative reduction in calories and sugar from taxed beverages, the absolute reductions were small in the post taxation period (2014–2016). To put into context our absolute calories and sugar reduction results, let us consider first, that the World health Organization (WHO) recommends the average daily sugar intake of an adult to be under 50 g of sugar (200 Kcal) [38], second, that in Mexico the average sugar intake is 60 g (238 Kcal), 19% above the WHO recommendation [7], and third, that 38% (23 g or 90 Kcal) of that sugar comes from taxed beverages [10]. From 2012 to 2016, total sugars from taxed beverage purchases decreased from 21 g to 16 g /capita/day (a decrease of 5 g or 20 kcal), which would result in a weight reduction of 0.9 kg/capita based on the modeling analyses conducted in Mexican population by Basto-Abreu et al. [39]. However, our results showed that in the second year post tax (2015) when we observed the largest decrease in sugar, the decline was of only 2 g (8Kcal). Thus, to achieve even more meaningful declines in the population sugar intake from SSBs (i.e. a 50% reduction in sugars from SSBs [roughly − 50.6 kcal from sugars] [39]), it is important that the SSBs tax is updated to consider inflation or increased to 2 MXN peso/liter (≈20%) as has been recommended to further curb SSBs purchases and intake, and achieve meaningful reductions in overweight, obesity and NCDs [40–44]. Furthermore, the SSBs tax should be accompanied by other obesity prevention strategies including restrictions in unhealthy food and beverage marketing, a clear front-of pack labeling system, norms to ensure the availability of healthy food and beverages in schools, and point-of-place interventions at retailers where most SSBs are purchased.

Compared with the 8% reduction in purchases of taxed beverage two years after the implementation of the SSBs tax reported by Colchero et al. [9], the 17% reduction we present for the same period in this study is comparatively larger. However, there are important points to consider before doing a direct comparison between studies. First, Colchero et al. results show the observed changes in purchases relative to what would be expected if the tax had not been implemented (counterfactual comparison). There was a downward trend from the two pre-taxation years (2012 and 2013) in the purchases of taxed beverages. Colchero et al. extrapolated this pre-tax trend through 2014 and 2015 to estimate their counterfactual and compared it to the observed change. This meant that part of the total change observed between 2013 and 2014 was attributed to the pre-tax trend. However, in this study we estimated the total change between years without comparing it to a counterfactual or accounting for a pre-tax trend, as we lacked enough time periods to provide a consistent counterfactual up to 3 years post tax [45]. Supposing that Colchero et al. had presented trend results as we do in this study, then the relative decline between the last pre-tax year and the first post-tax year (2013 to 2014) would have been − 16%, and the decline from the first to the second post-tax year (2014 to 2015) would have been − 12%, closely resembling our findings. Another difference between this and previous analyses is that we used data with complete reconstitution of all dry beverages (i.e. instant coffees), whereas Colchero et al. data included the reconstituted volume for dry sugary drinks powders only. This difference in reconstitution methodology affected the total volume reported in both studies (e.g., we report 50 mL more in the 2012 volume of taxed beverage purchases compared to Colchero et al.), however the purchasing trend was not affected as explained above.

This study shows that the relative change in volume of taxed beverages is greater than the change in calories and sugar. The fact that some taxed beverages contained a combination of caloric and non-caloric sweeteners might explain these findings. Beverages with any added sugar content would be taxed; however, those also sweetened with non-caloric sweeteners would have a limited absolute amount of sugar to reduce, which would reflect as a smaller relative change in caloric and sugar content over time. Moreover, the content of sugar may vary across taxed products, and a large decline in purchases of products with low sugar content would translate in great relative declines of volume but mild relative declines of calories and sugar. However, it is important to highlight that calories and sugar did decrease from the pre tax years (2012–2013) to the post tax years (2014–2016), and although the absolute declines are small in average, declines can potentially be larger among higher consumers and have an important impact in the health outcomes of the population at most risk [12]. Furthermore, one of the key motivators for our research was that people could have switched to untaxed beverages with high caloric content (e.g. 100% fruit juices) after the implementation of the tax, and even if we had observed a reduction in the volume of taxed beverages, we could have found an increase in calories. Although such results were unlikely since most untaxed beverages have few calories, the fact that our packages beverages data did not reflect an unintended substitution with other high caloric beverages reassures the success of the tax in decreasing purchases of sugary drinks.

Because we adjusted for the selectivity associated with households’ decision of where to shop, the variation in purchasing choices we observed across store-types (i.e. the sharp change in purchases observed at supermarkets in the first year post tax (2014)) are not likely to be explained by sociodemographic differences. Rather, the shopping occasion might be driving these differences in results, as supermarket shopping is usually intended for stock-up trips that can involve purchasing of larger quantities of products and involve planning ahead, whereas purchases at smaller stores (i.e. convenience stores) are associated with impulsive purchases, probably of less healthy products [46, 47]. An alternative explanation for the differences in the purchases of taxed and untaxed beverages observed by store-type is the amount of alternative untaxed beverages available at different retailers. The product assortment at supermarkets is expected to be larger than at smaller stores, thus, a wider range of alternative drinks would be available for supermarket shoppers compared to convenience store shoppers, which would reduce the possibility of purchasing choices. Future studies stratified by SES are needed to understand the socioeconomic determinants of the tax effect on calories and sugar from beverage purchases and how this may differ across store types.

The lack of change in taxed beverage purchases at convenience stores across the study period is worth noting, as we expected it to follow the declines observed in the rest of the store-types. These findings could suggest that households are inflexible about their taxed beverages purchases at convenience stores, which could be single drink purchases for immediate consumption. However, our results might be explained by the absolute amount of purchases made at convenience stores, which are small compared to other store-types (i.e. traditional retailers and supermarkets). It is more likely for households’ purchases to be affected by the tax at store-types where they buy larger volumes, hence, purchases might be less impacted if households buy smaller amounts at convenience stores.

The significant increase in purchases of untaxed beverages at supermarkets, other retailers, and home-delivery suggests that households purchasing choices shifted towards healthier choices by choosing to shop at store-types where larger volumes of non-caloric beverages are typically found. Interestingly, the largest amount of energy and sugar from purchases of untaxed beverages, though small, was observed at wholesalers, suggesting that some untaxed beverages with caloric and sugar content (i.e. 100% fruit juice), are highly purchased at wholesalers or price clubs.

This research has some limitations. One key consideration is that in this study we used the same nutritional profile for a given product across the entire time-period (both pre-tax and post-tax). The nutrition facts panel and ingredients data were collected one-year post-tax, and then these nutritional facts panel data were linked to purchases across the entire time period (pre- and post- tax). This means that our calorie and sugar estimates are likely to be conservative, because they do not reflect any reformulation (e.g., reductions in sugar to avoid the tax) that may have occurred. If beverages were reformulated by substituting sugar with non-caloric sweeteners, we would expect the decreases in calories and sugars from taxed beverage purchases to be larger than what our study found. Although the evaluation of reformulation was beyond the scope of this study, we observed an increase in water and diet products in the Nielsen CPS dataset over our study period, which can suggest the industry introduction of new products or reformulation as well as consumer purchasing changes. Since the reformulation of beverages might have shifted their nutritional profile and taxation status, future research should look at the annual variation on the taxation status of beverages as well as changes in the nutritional content of products to obtain refined understanding of the effect of the tax on the manufacturing, purchasing, and intake of the targeted beverages in every post taxation year.

Another limitation is that the Nielsen CPS only captures products with barcodes purchased at retail food outlets. Thus, home-prepared drinks with sugar (i.e. aguas frescas), concentrates normal bar-coded containers of taxed beverages bought at restaurants, and sugary drinks purchased from street vendors were not captured. Beverages sold by vendors and restaurants (such as concentrates or bottles) were taxed but not accounted for in our study. Considering that in Mexico 22% of food and beverages are consumed away-from-home, that 54% of Mexicans consume drinks from street vendors, and that 21% of the energy from SSB is consumed away-from-home [48–50], this lack of information on away-from-home beverages and other missing beverages affects our results on the net impact of taxation on purchases of packaged beverages. Assuming that the taxation of sugary drinks also declined purchases of beverages at the sources unaccounted for in this study, it is possible that our findings are bias towards underestimation. However, if the tax is not having an effect on away-from-home purchases, these results would overestimate their net effect.

Finally, although purchasing data can provide reasonable estimates of consumers overall diet quality [13], an important limitation of the Nielsen CPS is that it collects purchasing data and not intake. Thus, we cannot infer or verify how much of the product was consumed, how the purchased beverages were distributed within the household and whether all household members had the same intake.

Our data has several strengths as well. The Nielsen CPS purchasing data provides information of the beverages that were purchased at a store-specific level, which is often unavailable from other data sources, allowing us to estimate changes by store-type. Also, these data are collected throughout the year so seasonality variation is not a concern. Furthermore, having the nutritional information linked at the barcode level and reviewed by trained nutritionists for each product allowed us to have accurate assignation of taxation status as well as to estimate changes in calories and sugar over time using robust statistical analyses.

## Conclusions

From 2012 to 2016, we observed significant reductions in volume, calories and sugar purchases of taxed beverages and increases in the volume of untaxed beverage purchases. The biggest declines observed in the volume, calories and sugars of taxed beverages occurred in the first and second year post-tax, and plateaued in the third year post-tax.

The highest purchases of SSBs were observed at traditional stores. The volume, calories and sugar of taxed beverage purchases decreased over time for most store-types, however, the greatest significant decline in volume, calories and sugars of taxed beverages was observed in supermarkets from the last pre-tax year (2013) to the first post-tax year (2014). Overall absolute changes in calories and sugars by store-type were small.

Although the Mexican tax on SSBs has lowered the purchases of sugary drinks 3 years after the tax implementation, to achieve larger changes towards positive purchasing choices that help in the prevention of obesity, we echo the recommendation of increasing the SSBs tax in Mexico to 2 MXN pesos/liter (20%) [40, 41]. Furthermore, it is crucial that the SSBs tax is accompanied by a set of policy actions including unhealthy food and beverage marketing restrictions, the implementation of a clear front-of pack labeling system, and norms for healthy food and beverages in schools, to shift the food supply and further reduce purchases of SSBs [51, 52]. Moreover, because interventions and policies in food stores have been used to increase their healthfulness, potentially improve households diet quality, and prevent obesity [16–18], we suggest the implementation of point-of purchase interventions aimed to modify purchasing choices at traditional retailers where most taxed products are acquired and at other store-types that were less responsive to the tax effect.

We expect these results to provide evidence that helps to understand the link between the Mexican food environment and households’ food purchases, aiding to improve and promote healthier purchasing choices.

## Supplementary information

**Additional file 1: Table S1.** Beverages categories available in The Nielsen Company’s Mexico Consumer Panel Services 2012–2016 by taxation status. Table containing the detailed description of beverage subgroups categorized into taxed and untaxed.

**Additional file 2: Table S2.** Sociodemographic characteristics of household in The Nielsen Company’s Mexico Consumer Panel Services 2012–2016. Table containing the sociodemographic characteristics of households from the Nielsen CPS from 2012 to 2016.

**Additional file 3: Table S3.** Unadjusted mean volume, kilocalories and total sugar of taxed and untaxed beverages purchased by of Nielsen CPS households overall and by store-type in 2012 and 2016. Table containing unadjusted means of volume, calories and sugar overall and by store-type in 2012 and 2016 from the Nielsen CPS.

**Additional file 4: Table S4.** Predicted mean volume, kilocalories and total sugar of taxed and untaxed beverages purchased by of Nielsen CPS households (per capita/day) from 2012 to 2016. Table containing the overall fixed effects predicted means of volume, calories and sugar, and the absolute and relative differences between previous years from 2012 to 2016.

**Additional file 5: Table S5.** Predicted mean volume, kilocalories and total sugars (per capita/day) of taxed and untaxed beverages purchased by Nielsen CPS households from 2012 to 2016 by store-type. Table containing the store-type inverse probability weighted and fixed effects predicted means and the absolute and relative differences between previous years for volume, calories and sugar from 2012 to 2016.

## Data Availability

The data that support the findings of this study are available from The Nielsen Company (Mexico) but restrictions apply to the availability of these data, which were used under license for the current study, and so are not publicly available. Data are however available from the authors upon reasonable request and with permission of The Nielsen Company (Mexico).

## Abbreviations

INFORMAS: International Network for Food and Obesity/non-communicable diseases Research, Monitoring and Action Support
INSP: Instituto Nacional de Salud Pública (Mexican National Institute of Public Health)
MxNFP: Mexican Nutrition Fact Panel
Nielsen CPS: The Nielsen Company’s Mexico Consumer Panel Services
PLA: Product Launch Analytics
SES: Socioeconomic status
SSBs: Sugar-sweetened beverages
WHO: World Health Organization
