# Supplementary material for: The caloric and sugar content of beverages purchased at different store-types changed after the sugary drinks taxation in Mexico
Source: Int J Behav Nutr Phys Act. 2019 Nov 12;16:103. doi: 10.1186/s12966-019-0872-8 (PMC6849184; doi:10.1186/s12966-019-0872-8)
Supplement: Supplementary file 1 — Additional file 1: Table S1. Beverages categories available in The Nielsen Company’s Mexico Consumer Panel Services 2012–2016 by taxation status. Table containing the detailed description of beverage subgroups categorized into taxed and untaxed. [file 12966_2019_872_MOESM1_ESM.docx]

| **Additional file 1: Table S1. Beverages categories available in The Nielsen Company’s Mexico Consumer Panel Services 2012-2016 by taxation status** | | |
| --- | --- | --- |
| **Taxation status*** | **Categorization** | **Products included** |
| Taxed | Sugar sweetened sodas | Sodas with added sugar |
|  | Non-carbonated sugar sweetened beverages | Sugar-sweetened beverages (others than soda) including industrialized flavored waters, tea, soy and non-dairy base beverages, atole and coffee. |
|  | Juices from concentrate | Sweetened juices |
|  | Other sugary beverages | Soy and non-dairy base beverages, coffee |
| Untaxed | Artificially Sweetened Sodas | Sodas |
|  | Non-carbonated artificially sweetened beverages | Industrialized flavored water and tea without added sugar or with non-caloric sweeteners |
|  | 100% Juices | 100% fruit or vegetable juices |
|  | Water | Plain and sparkling water |
| *Mexican legislation taxes with 1 peso/liter all beverages with added sugar. A team of Mexican registered dieticians reviewed each product and assigned it into a beverage and tax category following the law definitions.  Note that The Nielsen CPS did not collect information on purchases of dairy products and beer consistently during 2012-2016, thus, we excluded dairy products and beer from the analyses for this study. | | |
