# Supplementary material for: The caloric and sugar content of beverages purchased at different store-types changed after the sugary drinks taxation in Mexico
Source: Int J Behav Nutr Phys Act. 2019 Nov 12;16:103. doi: 10.1186/s12966-019-0872-8 (PMC6849184; doi:10.1186/s12966-019-0872-8)
Supplement: Supplementary file 2 — Additional file 2: Table S2. Sociodemographic characteristics of household in The Nielsen Company’s Mexico Consumer Panel Services 2012–2016. Table containing the sociodemographic characteristics of households from the Nielsen CPS from 2012 to 2016. [file 12966_2019_872_MOESM2_ESM.docx]

| **Additional file 2: Table S2. Sociodemographic characteristics of household in The Nielsen Company’s Mexico Consumer Panel Services 2012-2016.** | | | | | |  |
| --- | --- | --- | --- | --- | --- | --- |
| Year | **2012** | **2013** | **2014** | **2015** | **2016** | |
| Number of households | 5813 | 5775 | 5656 | 5493 | 5606 | |
| Number of household-year observations | 69343 | 69092 | 67505 | 65125 | 67122 | |
| **Socioeconomic level (%)** |  |  |  |  |  | |
| Low | 20 | 23 | 25 | 25 | 18 | |
| Middle | 58 | 53 | 51 | 52 | 57 | |
| High | 22 | 24 | 24 | 23 | 24 | |
| **Household size (%)** |  |  |  |  |  | |
| 2-3 | 16 | 15 | 14 | 14 | 14 | |
| 4-5 | 43 | 42 | 41 | 41 | 41 | |
| 6-7 | 22 | 23 | 23 | 22 | 22 | |
| ≥8 | 19 | 21 | 23 | 24 | 22 | |
| **Number of children in household (0-19 y) (%)** |  |  |  |  |  | |
| 0-1 | 35 | 39 | 39 | 41 | 37 | |
| 2-3 | 46 | 44 | 44 | 42 | 43 | |
| 4-5 | 14 | 13 | 12 | 12 | 14 | |
| ≥6 | 6 | 5 | 5 | 5 | 6 | |
| Source: Authors’ own analyses and calculations based on data from Nielsen through its Mexico Consumer Panel Service (CPS), for the beverage categories for January 2012 – December 2016. The Nielsen Company, 2016. Nielsen is not responsible for and had no role in preparing the results reported herein. SES classification is based on the socioeconomic index provided by Nielsen CPS that includes seven household assets (number of rooms, type of floor, number of bathrooms, shower, gas range, number of light bulbs and number of cars), and the education level of the head of the household . All means and proportions are weighted using projection factors provided by Nielsen CPS to represent populations in areas with more than 50,000 inhabitants. | | | | | | |
