# Supplementary material for: The caloric and sugar content of beverages purchased at different store-types changed after the sugary drinks taxation in Mexico
Source: Int J Behav Nutr Phys Act. 2019 Nov 12;16:103. doi: 10.1186/s12966-019-0872-8 (PMC6849184; doi:10.1186/s12966-019-0872-8)
Supplement: Supplementary file 3 — Additional file 3: Table S3. Unadjusted mean volume, kilocalories and total sugar of taxed and untaxed beverages purchased by of Nielsen CPS households overall and by store-type in 2012 and 2016. Table containing unadjusted means of volume, calories and sugar overall and by store-type in 2012 and 2016 from the Nielsen CPS. [file 12966_2019_872_MOESM3_ESM.docx]

| **Additional file 3: Table S3. Unadjusted mean volume, kilocalories and total sugar of taxed and untaxed beverages purchased by of Nielsen CPS households overall and by store-type in 2012 and 2016.** | | | | | | | | | | | | |
| --- | --- | --- | --- | --- | --- | --- | --- | --- | --- | --- | --- | --- |
|  | **Volume (ml/capita/day)** | | | | **Kilocalories (Kcal/capita/day)** | | | | **Total sugar (g/capita/day)** | | | |
| Taxation status | **Taxed** | | **Untaxed** | | **Taxed** | | **Untaxed** | | **Taxed** | | **Untaxed** | |
| Year | **2012** | **2016** | **2012** | **2016** | **2012** | **2016** | **2012** | **2016** | **2012** | **2016** | **2012** | **2016** |
|  |  |  |  |  |  |  |  |  |  |  |  |  |
| Overall | 264 (261,267) | 192 (189,194) | 800 (791,809) | 904 (893,915) | 80 (79,81) | 69 (68,70) | 1 (1,1) | 1(0,1) | 19 (19,20) | 17 (17,17) | 0.1 (0.1,0.1) | 0.1 (0.1,0.1) |
|  |  |  |  |  |  |  |  |  |  |  |  |  |
| **Store-type** |  |  |  |  |  |  |  |  |  |  |  |  |
| Convenience Stores | 5 (4,5) | 6 (5,6) | 7 (6,7) | 11 (10,12) | 2 (2,2) | 2 (2,2) | 0 (0,0) | 0 (0,0) | 0 (0,0) | 1 (0,1) | 0 (0,0) | 0 (0,0) |
| Supermarkets | 59 (58,60) | 31 (31,32) | 37 (36,38) | 59 (57,60) | 9 (9,9) | 7 (7,8) | 0 (0,0) | 1 (1,1) | 2 (2,2) | 2 (2,2) | 0 (0,0) | 0 (0,0) |
| Wholesalers | 4 (4,5) | 2 (2,2) | 6 (6,7) | 7 (7,8) | 1 (1,1) | 1 (0,1) | 0 (0,0) | 0 (0,0) | 0 (0,0) | 0 (0,0) | 0 (0,0) | 0 (0,0) |
| Traditional stores | 190 (188,192) | 148 (146,151) | 138 (133,143) | 148 (144,152) | 67 (66,68) | 57 (56,58) | 0 (0,0) | 0 (0,0) | 16 (16,17) | 14 (14,14) | 0 (0,0) | 0 (0,0) |
| Others | 5 (4,5) | 3 (3,3) | 147 (142,152) | 156 (151,161) | 1 (1,1) | 1 (1,1) | 0 (0,0) | 0 (0,0) | 0 (0,0) | 0 (0,0) | 0 (0,0) | 0 (0,0) |
| Home-delivery | 1 (1,1) | 1 (1,2) | 465 (457,473) | 523 (513,533) | 0 (0,0) | 1 (0,1) | 0 (0,0) | 0 (0,0) | 0 (0,0) | 0 (0,0) | 0 (0,0) | 0 (0,0) |
| Source: Authors’ own analyses and calculations based on data from Nielsen through its Mexico Consumer Panel Service (CPS), for the beverage categories for January 2012 – December 2016. The Nielsen Company, 2016. Nielsen is not responsible for and had no role in preparing the results reported herein. All means are weighted using projection factors provided by Nielsen CPS to represent populations in areas with more than 50,000 inhabitants. | | | | | | | | | | | | |
