# Supplementary material for: The caloric and sugar content of beverages purchased at different store-types changed after the sugary drinks taxation in Mexico
Source: Int J Behav Nutr Phys Act. 2019 Nov 12;16:103. doi: 10.1186/s12966-019-0872-8 (PMC6849184; doi:10.1186/s12966-019-0872-8)
Supplement: Supplementary file 4 — Additional file 4: Table S4. Predicted mean volume, kilocalories and total sugar of taxed and untaxed beverages purchased by of Nielsen CPS households (per capita/day) from 2012 to 2016. Table containing the overall fixed effects predicted means of volume, calories and sugar, and the absolute and relative differences between previous years from 2012 to 2016. [file 12966_2019_872_MOESM4_ESM.docx]

| **Additional file 4: Table S4. Predicted mean volume, kilocalories and total sugar of taxed and untaxed beverages purchased by of Nielsen CPS households (per capita/day) from 2012-2016.** | | | | | | | | |
| --- | --- | --- | --- | --- | --- | --- | --- | --- |
|  | **Taxed beverages** | | | | **Untaxed beverages** | | | |
|  | Year | Mean (95 %CI) | Absolute difference (95% CI) with previous year | Relative (%) difference with previous year | Year | Mean (95 %CI) | Absolute difference (95% CI) with previous year | Relative (%) difference with previous year |
| Volume (ml/capita/day) | 2012 | 286 (266,305) | - | - | 2012 | 781 (712,850) | - | - |
|  | 2013 | 260 (250,271) | -25 (-40,-10)* | -9% | 2013 | 811 (773,848) | 29 (-20,79) | 4% |
|  | 2014 | 211 (208,215) | -49 (-63,-35)* | -19% | 2014 | 904 (891,917) | 93 (42,144)* | 11% |
|  | 2015 | 181 (171,191) | -30 (-48,-13)* | -14% | 2015 | 872 (838,906) | -32 (-90,27) | -3% |
|  | 2016 | 178 (158,198) | -3 (-20,13) | -2% | 2016 | 909 (840,979) | 37 (-22,96) | 4% |
|  |  |  |  |  |  |  |  |  |
| Kilocalories (per capita/day) | 2012 | 84 (77,91) | - | - | 2012 | 0.9 (0.7,1.2) | - | - |
|  | 2013 | 79 (75,82) | -5 (-11,0) | -6% | 2013 | 0.9 (0.8,1.1) | 0 (-0.2,0.2) | -2% |
|  | 2014 | 74 (72,75) | -5 (-8,-2)* | -6% | 2014 | 1.3 (1.3,1.4) | 0.4 (0.2,0.6)* | 44% |
|  | 2015 | 66 (62,69) | -8 (-14,-2)* | -11% | 2015 | 1.2 (.01,1.3) | -0.2 (-0.4,0.1) | -12% |
|  | 2016 | 66 (59,73) | 0 (-6,6) | 0% | 2016 | 1.0 (0.7,1.3) | -0.2 (-0.4,0.1) | -14% |
|  |  |  |  |  |  |  |  |  |
| Total sugars (per capita/day) | 2012 | 21 (19,22) | - | - | 2012 | 0.14 (0.08,0.19) | - | - |
|  | 2013 | 19 (18,20) | -1 (-3,1) | -6% | 2013 | 0.13 (0.11,0.16) | 0 (-0.04,0.04) | -2% |
|  | 2014 | 18 (18,18) | -1 (-2,0)* | -6% | 2014 | 0.13 (0.12,0.14) | -0.01 (-0.04,0.03) | -5% |
|  | 2015 | 16 (15,17) | -2 (-3,-1)* | -11% | 2015 | 0.11 (0.09,0.13) | -0.02 (-0.06,0.02) | -14% |
|  | 2016 | 16 (14,18) | 0 (-2,1) | -1% | 2016 | 0.1 (0.05,0.16) | 0 (-0.05,0.04) | -4% |
| Source: Authors’ own analyses and calculations based on data from Nielsen through its Mexico Consumer Panel Service (CPS), for the beverage categories for January 2012 – December 2016. The Nielsen Company, 2016. Nielsen is not responsible for and had no role in preparing the results reported herein. Volume, kilocalories and total sugar means of taxed and untaxed beverage purchases obtained using fixed effect models adjusted by socioeconomic index, household size and composition, minimum wage, unemployment rate and consumer price index, and weighted to be representative of populations in areas with more than 50 000 inhabitants. *p-value <0.05 comparing with previous year using the Bonferroni method to account for multiple comparisons. | | | | | | | | |
