# Supplementary material for: The caloric and sugar content of beverages purchased at different store-types changed after the sugary drinks taxation in Mexico
Source: Int J Behav Nutr Phys Act. 2019 Nov 12;16:103. doi: 10.1186/s12966-019-0872-8 (PMC6849184; doi:10.1186/s12966-019-0872-8)
Supplement: Supplementary file 5 — Additional file 5: Table S5. Predicted mean volume, kilocalories and total sugars (per capita/day) of taxed and untaxed beverages purchased by Nielsen CPS households from 2012 to 2016 by store-type. Table containing the store-type inverse probability weighted and fixed effects predicted means and the absolute and relative differences between previous years for volume, calories and sugar from 2012 to 2016. [file 12966_2019_872_MOESM5_ESM.docx]

| **Additional file 5: Table S5. Predicted mean volume, kilocalories and total sugars (per capita/day) of taxed and untaxed beverages purchased by Nielsen CPS households from 2012-2016 by store-type.** | | | | | | | | | | | | |
| --- | --- | --- | --- | --- | --- | --- | --- | --- | --- | --- | --- | --- |
|  | **Volume** | | | | | | **Kilocalories** | | | | | |
|  | Taxed | | | Untaxed | | | Taxed | | | Untaxed | | |
|  | Mean (95 %CI) | Absolute difference (95% CI) with previous year | Relative (%) difference with previous year | Mean (95 %CI) | Absolute difference (95% CI) with previous year | Relative (%) difference with previous year | Mean (95 %CI) | Absolute difference (95% CI) with previous year | Relative (%) difference with previous year | Mean (95 %CI) | Absolute difference (95% CI) with previous year | Relative (%) difference with previous year |
| **Convenience stores** |  |  |  |  |  |  |  |  |  |  |  |  |
| 2012 | 29 (20,38) | - | - | 66 (37,94) | - | - | 10 (7,14) | - | - | 0.2 (0,0.4) | - | - |
| 2013 | 28 (24,33) | -1 (-9,6) | -4% | 60 (46,74) | -6 (-29,16) | -10% | 10 (8,11) | -1 (-3,2) | -5% | 0.2 (0.2,0.3) | 0.02 (-0.2,0.2) | 10% |
| 2014 | 26 (24,28) | -2 (-9,4) | -8% | 56 (52,60) | -3 (-22,16) | -6% | 10 (9,10) | 0 (-3,2) | -1% | 0.3 (0.2,0.3) | 0.03 (-0.1,0.1) | 13% |
| 2015 | 25 (20,30) | -1 (-9,7) | -3% | 39 (24,54) | -17 (-42,7) | -31% | 9 (7,11) | -1 (-4,2) | -6% | 0.2 (0.1,0.3) | -0.05 (-0.2,0.1) | -17% |
| 2016 | 27 (17,37) | 2 (-6,10) | 9% | 29 (-2,60) | -10 (-34,14) | -25% | 10 (6,14) | 1 (-2,4) | 13% | 0.1 (-0.1,0.3) | -0.08 (-0.3,0.1) | -36% |
| **Supermarkets** |  |  |  |  |  |  |  |  |  |  |  |  |
| 2012 | 103 (95,111) | - | - | 23 (13,34) | - | - | 14 (12,16) | - | - | 0.6 (0.4,0.8) | - | - |
| 2013 | 88 (84,92) | -15 (-21,-9)* | -15% | 43 (37,48) | 19 (11,27)* | 82% | 13 (12,14) | -1 (-2,1) | -5% | 0.7 (0.6,0.8) | 0.09 (-0.1,0.2) | 16% |
| 2014 | 48 (47,50) | -40 (-46,-34)* | -45% | 88 (86,89) | 45 (37,53)* | 106% | 11 (11,11) | -2 (-3,-1)* | -16% | 1.1 (1,1.1) | 0.41 (0.3,0.5)* | 61% |
| 2015 | 36 (32,41) | -12 (-19,-5)* | -25% | 98 (93,103) | 10 (2,19)* | 12% | 10 (9,11) | -1 (-3,0) | -10% | 1 (0.9,1.2) | -0.03 (-0.2,0.1) | -3% |
| 2016 | 33 (25,42) | -3 (-8,2) | -8% | 111 (100,123) | 13 (4,23)* | 14% | 10 (9,12) | 0 (-1,2) | 4% | 1 (0.8,1.2) | 0 (-0.2,0.2) | 0% |
| **Wholesalers** |  |  |  |  |  |  |  |  |  |  |  |  |
| 2012 | 68 (45,92) | - | - | 95 (45,145) | - | - | 18 (12,24) | - | - | 2.4 (0.9,3.9) | - | - |
| 2013 | 57 (45,69) | -11 (-30,7) | -17% | 109 (85,132) | 14 (-28,55) | 14% | 15 (12,19) | -3 (-8,2) | -15% | 2.2 (1.5,3.0) | -0.16 (-1.4,1.1) | -7% |
| 2014 | 39 (34,43) | -19 (-37,0) | -32% | 130 (122,138) | 21 (-11,53) | 19% | 11 (10,12) | -4 (-10,1) | -28% | 2.7 (2.3,3.1) | 0.48 (-0.7,1.6) | 21% |
| 2015 | 36 (24,48) | -2 (-21,16) | -6% | 138 (111,164) | 8 (-35,51) | 6% | 10 (6,13) | -1 (-7,4) | -13% | 1.9 (1.1,2.7) | -0.79 (-2.1,0.5) | -29% |
| 2016 | 38 (12,65) | 2 (-20,25) | 7% | 152 (95,208) | 14 (-33,62) | 10% | 10 (3,17) | 0 (-6,6) | 0% | 1.5 (-0.2,3.2) | -0.45 (-1.8,0.9) | -23% |
| **Traditional stores** |  |  |  |  |  |  |  |  |  |  |  |  |
| 2012 | 210 (196,223) | - | - | 143 (112,174) | - | - | 74 (69,80) | - | - | 0.1 (0.0,0.3) | - | - |
| 2013 | 196 (189,203) | -14 (-24,-3)* | -6% | 143 (127,159) | 0 (-23,23) | 0% | 70 (68,73) | -4 (-8,0) | -5% | 0.2 (0.1,0.2) | 0.04 (-0.1,0.1) | 31% |
| 2014 | 179 (177,182) | -17 (-26,-8)* | -9% | 153 (149,158) | 10 (-12,33) | 7% | 68 (67,69) | -2 (-6,1) | -3% | 0.3 (0.3,0.3) | 0.14 (0.1,0.2)* | 76% |
| 2015 | 161 (154,169) | -18 (-30,-5)* | -10% | 146 (129,162) | -8 (-33,17) | -5% | 63 (60,66) | -5 (-10,0) | -7% | 0.4 (0.3,0.5) | 0.09 (0.0,0.2) | 27% |
| 2016 | 165 (150,180) | 3 (-9,16) | 2% | 139 (106,172) | -7 (-32,18) | -5% | 66 (60,72) | 3 (-2,8) | 4% | 0.5 (0.4,0.6) | 0.09 (0.0,0.2) | 21% |
| **Others** |  |  |  |  |  |  |  |  |  |  |  |  |
| 2012 | 17 (11,23) | - | - | 396 (307,485) | - | - | 3 (1,5) | - | - | 0.2 (0.0,0.4) | - | - |
| 2013 | 13 (10,17) | -4 (-8,1) | -23% | 467 (418,516) | 71 (8,133)* | 18% | 3 (2,4) | 0 (-1,1) | -5% | 0.2 (0.1,0.2) | -0.04 (-0.2,0.1) | -22% |
| 2014 | 15 (13,17) | 2 (-3,7) | 14% | 569 (537,602) | 103 (48,157)* | 22% | 4 (3,4) | 1 (-1,3) | 43% | 0.2 (0.2,0.2) | 0.04 (-0.1,0.2) | 25% |
| 2015 | 10 (7,13) | -5 (-11,1) | -32% | 595 (545,644) | 25 (-81,131) | 4% | 4 (3,4) | 0 (-2,1) | -8% | 0.2 (0.1,0.3) | 0.01 (-0.1,0.2) | 4% |
| 2016 | 8 (1,14) | -3 (-8,3) | -27% | 693 (581,805) | 98 (4,192)* | 16% | 4 (2,6) | 0 (-1,2) | 8% | 0.2 (0.0,0.4) | 0.00(-0.1,0.1) | -2% |
| **Home-delivery** |  |  |  |  |  |  |  |  |  |  |  |  |
| 2012 | 5 (3,7) | - | - | 839 (766,912) | - | - | 2 (1,3) | - | - | 0.1 (0.1,0.2) | - | - |
| 2013 | 4 (3,6) | -1 (-2,1) | -12% | 875 (838,913) | 36 (-17,89) | 4% | 2 (1,2) | 0 (-1,0) | -16% | 0.1 (0.1,0.2) | -0.02 (-0.1,0) | -14% |
| 2014 | 4 (3,5) | -1 (-2,1) | -12% | 963 (947,978) | 87 (36,139)* | 10% | 2 (1,2) | 0 (-1,0) | -12% | 0.1 (0.1,0.1) | -0.03 (-0.1,0) | -25% |
| 2015 | 2 (1,3) | -2 (-4,0) | -44% | 970 (932,1007) | 7 (-59,73) | 1% | 1 (0,1) | -1 (-2,0) | -48% | 0.0 (-0.1,0) | -0.13 (-0.2,0)* | -136% |
| 2016 | 1 (-1,3) | -1 (-3,1) | -57% | 1037 (957,1117) | 67 (1,133)* | 7% | 0 (-1,1) | -1 (-1,0) | -67% | -0.1 (-0.2,0) | -0.08 (-0.1,0)* | 227% |

| **Cont. Supplemental table 5. Predicted mean volume, kilocalories and total sugars (per capita/day) of taxed and untaxed beverages purchased by Nielsen CPS households from 2012-2016 by store-type.** | | | | | | |
| --- | --- | --- | --- | --- | --- | --- |
|  | **Total sugar** | | | | | |
|  | Taxed | | | Untaxed | | |
|  | Mean (95 %CI) | Absolute difference (95% CI) with previous year | Relative (%) difference with previous year | Mean (95 %CI) | Absolute difference (95% CI) with previous year | Relative (%) difference with previous year |
| **Convenience stores** |  |  |  |  |  |  |
| 2012 | 2 (2,3) | - | - | 0.02 (-0.02,0.06) | - | - |
| 2013 | 2 (2,3) | 0 (-1,1) | -5% | 0.03 (0.01,0.04) | 0.01 (-0.03,0.05) | 61% |
| 2014 | 2 (2,3) | 0 (-1,1) | -1% | 0.03 (0.02,0.03) | 0.00 (-0.02,0.02) | 8% |
| 2015 | 2 (2,3) | 0 (-1,1) | -6% | 0.03 (0.01,0.05) | 0.00 (-0.03,0.03) | 8% |
| 2016 | 2 (2,3) | 0 (0,1) | 13% | 0.03 (-0.01,0.08) | 0.00 (-0.03,0.04) | 13% |
| **Supermarkets** |  |  |  |  |  |  |
| 2012 | 3 (3,3) | - | - | 0.12 (0.08,0.16) | - | - |
| 2013 | 3 (3,3) | 0 (0,0) | -1% | 0.12 (0.10,0.14) | 0.00(-0.03,0.03) | 0% |
| 2014 | 3 (2,3) | 0 (-1,0)* | -16% | 0.11 (0.11,0.12) | 0.00 (-0.03,0.02) | -4% |
| 2015 | 2 (2,3) | 0 (-1,0) | -8% | 0.10 (0.08,0.12) | -0.01 (-0.05,0.02) | -10% |
| 2016 | 2 (2,3) | 0 (0,1) | 6% | 0.10 (0.06,0.14) | 0.00 (-0.03,0.03) | 2% |
| **Wholesalers** |  |  |  |  |  |  |
| 2012 | 3 (2,5) | - | - | 0.51 (0.24,0.77) | - | - |
| 2013 | 3 (2,4) | 0 (-1,1) | -9% | 0.38 (0.25,0.51) | -0.13 (-0.34,0.09) | -25% |
| 2014 | 2 (2,3) | -1 (-2,1) | -22% | 0.34 (0.27,0.42) | -0.04 (-0.25,0.18) | -10% |
| 2015 | 2 (1,3) | 0 (-1,1) | -8% | 0.11 (0.00,0.25) | -0.24 (-0.46,-0.01)* | -69% |
| 2016 | 2 (1,4) | 0 (-1,1) | 4% | 0.02 (0.00,0.27) | -0.13 (-0.37,0.11) | -120% |
| **Traditional stores** |  |  |  |  |  |  |
| 2012 | 18 (17,20) | - | - | 0.01 (-0.02,0.03) | - | - |
| 2013 | 17 (17,18) | -1 (-2,0) | -5% | 0.02 (0.01,0.03) | 0.01 (-0.01,0.03) | 116% |
| 2014 | 17 (17,17) | -1 (-1,0) | -3% | 0.03 (0.02,0.03) | 0.01 (-0.01,0.03) | 60% |
| 2015 | 16 (15,16) | -1 (-3,0)* | -8% | 0.05 (0.04,0.06) | 0.02 (0.00,0.04)* | 77% |
| 2016 | 16 (15,18) | 0 (-1,2) | 3% | 0.07 (0.05,0.10) | 0.03 (0.00,0.04)* | 54% |
| **Others** |  |  |  |  |  |  |
| 2012 | 1 (0,1) | - | - | 0.02 (0.00,0.03) | - | - |
| 2013 | 1 (0,1) | 0 (0,0) | -2% | 0.01 (0.00,0.02) | -0.01 (-0.02,0.01) | -34% |
| 2014 | 1 (1,1) | 0 (0,1) | 51% | 0.02 (0.01,0.02) | 0.01 (-0.01,0.02) | 54% |
| 2015 | 1 (1,1) | 0 (0,0) | -6% | 0.02 (0.01,0.02) | 0.00 (-0.01,0.01) | -6% |
| 2016 | 1 (1,1) | 0 (0,0) | 10% | 0.01 (0.00,0.03) | 0.00 (-0.02,0.01) | -11% |
| **Home-delivery** |  |  |  |  |  |  |
| 2012 | 1 (0,1) | - | - | 0.03 (0.01,0.1) | - | - |
| 2013 | 0 (0,1) | 0 (0,0) | -16% | 0.03 (0.02,0) | 0 (-0.02,0) | -14% |
| 2014 | 0 (0,0) | 0 (0,0) | -11% | 0.02 (0.01,0) | -0.01 (-0.02,0) | -25% |
| 2015 | 0 (0,0) | 0 (0,0) | -49% | -0.01 (-0.02,0) | -0.03 (-0.06,0)* | -137% |
| 2016 | 0 (0,0) | 0 (0,0) | -67% | -0.02 (-0.05,0) | -0.02 (-0.03,0)* | 221% |
| Source: Authors’ own analyses and calculations based on data from Nielsen through its Mexico Consumer Panel Service (CPS), for the beverage categories for January 2012 – December 2016. The Nielsen Company, 2016. Nielsen is not responsible for and had no role in preparing the results reported herein. Volume, kilocalories and total sugar means of taxed and untaxed beverage purchases obtained using fixed effect models weighted with store-specific inverse probability weights and adjusted by socioeconomic index, household size and composition, minimum wage, unemployment rate and consumer price index, and weighted to be representative of populations in areas with more than 50 000 inhabitants. *Difference between years with a p-value <0.05 comparing with previous year using the Bonferroni method to account for multiple comparisons. | | | | | | |
